# Australian nursing students’ perception, knowledge, and attitude towards oral healthcare of older people and associated factors: a national cross-sectional survey

**DOI:** 10.1186/s12912-023-01366-x

**Published:** 2023-06-05

**Authors:** Vandana Bhagat, Ha Hoang, Leonard A. Crocombe, Lynette R. Goldberg

**Affiliations:** 1 Centre for Rural Health, University of Tasmania, Tasmania, Australia; 2 Department of Rural Health, La Trobe University, Melbourne, Australia; 3 Wicking Dementia Research and Education Centre, University of Tasmania, Tasmania, Australia

**Keywords:** Oral health, Oral care, Older people, Nursing, Attitudes, Education, Understanding, Knowledge, Curricula

## Abstract

**Background:**

The oral health of many older Australians is poor and associated with many systemic health problems. However, nurses often have a limited understanding of the importance of oral healthcare for older people. This study aimed to investigate Australian nursing students’ perception, knowledge, and attitude toward providing oral healthcare for older people and associated factors.

**Methods:**

A cross-sectional study was conducted among final year nursing students studying at accredited nursing programs using an online self-reported 49-item survey. The data were analysed using univariate and bivariate analysis (*t*-test, ANOVA, Spearman’s correlation test).

**Results:**

A total of 416 final-year nursing students from 16 accredited programs in Australia completed the survey. Mean scores showed that more than half of the participants felt they lacked confidence (55%, n = 229) and had limited knowledge about oral healthcare for older people (73%, n = 304); however, their attitude towards providing such care was favourable (89%, n = 369). A positive correlation was found between students’ confidence in delivering oral healthcare to older people and their perceived knowledge (*r* = 0.13, *p* < 0.01). Results revealed a statistically significant positive association between students’ experience in providing oral healthcare to older people and students’ perception (*t* = 4.52, *p* < 0.001), knowledge (*t* = 2.87, *p* < 0.01), and attitude (*t* = 2.65, *p* < 0.01) mean scores in such care. Nearly 60% (n = 242) of participants received education/training in oral healthcare for older people at university, but this was often for less than one hour. Around 56% (n = 233) believed that the current nursing curriculum did not prepare them to provide effective oral healthcare to older people.

**Conclusion:**

Findings suggested a need for nursing curricula to be revised to include oral health education and clinical experience. Knowledge of evidence-based oral healthcare by nursing students may improve the quality of oral healthcare for older people.

## Background

Oral health is essential to people’s general health and quality of life [1]. However, the oral health of many older Australians is poor [2]. More than 60% of Australians over 75 years of age suffer from gum disease, and one in three has lost all of their teeth [3]. Oral candidiasis, denture stomatitis, denture-irritation hyperplasia, and traumatic ulceration are all conditions that can develop as a result of oral neglect [4]. Poor oral health can disturb chewing and swallowing, affecting nutritional intake [3]. Gum diseases also have a strong link to respiratory problems [5, 6]. Recent evidence shows that adults with poor oral health showed more severe complications and delayed recovery from COVID-19 [7, 8].

Older people’s ability to take care of their oral health can deteriorate due to a decline in cognition and physical function [9]. Furthermore, oral health problems of older people with dementia can go undiagnosed and untreated as they may be less likely to complain [4]. Therefore, primary healthcare professionals need to collaborate to implement evidence-based oral healthcare, including appropriate dental referrals for early intervention [10]. Nurses play a crucial role in the delivery of healthcare services to older people who need assistance [11]. Nurses with knowledge, skills, and a positive attitude towards providing oral healthcare to older people are essential for older people’s health and well-being [9]. For nursing practice, oral healthcare entails ensuring daily oral hygiene, being able to complete an oral health screening, participating in the development of individualised oral care plans and adhering to them, and ongoing collaboration with dental, medical, and allied health professionals for the purpose of coordinating care for older people [12].

Oral health is clearly linked to general health [1, 6–8], yet oral health care is often a low priority among primary health care providers, including nurses [11]. This lack of priority can be due to under presentation of oral health content in nursing curricula. There were only a few studies that examined nursing students’ attitudes and knowledge on oral health, and they were carried out in the USA, Turkey, Japan, New Zealand, and Australia [13–17]. It has been found that nursing students often lack oral healthcare understanding. Geographical location affects people’s views on oral hygiene practices. Most of the studies used a convenience sample of nursing students from a single university, so results were not representative of the whole population. The knowledge and attitudes of nursing students concerning oral healthcare of older people were not assessed in the earlier investigations in Australia [16, 18]. Therefore, this study aimed to assess nursing students’ knowledge of and attitudes towards oral healthcare of older people. This would provide a baseline data set from which to build oral health educational models for nurses to deliver effective oral healthcare services to older people. Incorporating oral healthcare into nursing curricula will increase nurses’ knowledge of oral healthcare and its relevance to overall health outcomes [9].

## Methodology

### Operational terms used in this paper

- Perception of self-efficacy: Opinions of nursing students about their ability to provide effective oral healthcare for older people [19].
- Knowledge: Nursing students’ awareness and understanding of essential components in providing oral healthcare for older people [20].
- Attitude: An attribute expressing nursing students’ beliefs, feelings, and interest in providing oral healthcare for older people [21].

### Research questions

RQ 1: What is the perception of final-year undergraduate nursing students in Australia regarding their self-efficacy, knowledge, and attitude towards providing oral healthcare to older people?

RQ 2: What is the correlation between nursing students’ perception of self-efficacy to provide oral healthcare to older people and their knowledge of oral healthcare?

RQ 3: What factors are associated with nursing students’ perception, knowledge, and attitude toward providing oral healthcare for older people?

### Research design

A descriptive cross-sectional study using a self-report online survey was conducted from January to March 2021. The advantages of the cross-sectional survey are that it can reach a large number of respondents at a low cost and more efficiently compared to other data collection methods [22, 23]. Moreover, quantitative research is suitable for measuring and analysing relationships between numerous variables in a single study [24].

### Research setting

This study was conducted with undergraduate nursing students at Australian universities delivering a Bachelor of Nursing program accredited by the Australian Nursing and Midwifery Accreditation Council (ANMAC). ANMAC is an independent accrediting authority and acts as the nation’s gatekeeper in maintaining the quality of nursing curriculum design and the professional attributes necessary to become a registered nurse in Australia [25].

### Participants and sample size determination

This study recruited final-year undergraduate nursing students aged 18 years or older from 16 Australian universities that agreed to participate in this study. Final-year nursing students were chosen as they were expected to be exposed to older people’s oral healthcare needs in their curriculum or through clinical placements.

*Sample size determination*: Using a cross-sectional study formula [26], sample size was determined as below:-.

$$n = \frac{{Z_{(1 - \frac{\alpha }{2})}^2p(1 - p)}}{{{d^2}}} = \frac{{{{1.96}^2} \times 0.6 \times (1 - 0.6)}}{{{{0.05}^2}}} \approx 368$$

Where $p=$ 0.6, which is the expected proportion of the population based on a previous study [13].

$d=0.05$ which is the desired level of absolute precision

$Z=1.96$ is the specified absolute precision (standard normal variate)

and $n=$sample size.

### Data collection instrument and pilot study

A self-administered online survey was used to collect the data. The survey was developed based on a review of relevant studies and previously published survey instruments [27, 28]. Modifications to the published instruments were undertaken to fit all the objectives of the proposed study. The 49-item survey was organised into seven sections: (i) demographic information; (ii) experience providing oral healthcare for older people; (iii) perception of self-efficacy; (iv) knowledge; (v) attitude; (vi) personal oral health behaviour; (vii) open-ended questions to identify strategies to strengthen oral health education and clinical preparation. Sections (iv), (v), and (vi) featured 5-point Likert-type scales.

Pilot testing of the survey with recent graduates or those students about to graduate with a Bachelor of Nursing degree in Australia showed satisfactory validity and reliability of the survey [19]. The Cronbach’s alpha of items in the perception, knowledge, and attitude sections of the survey was 0.82, 0.76, and 0.60, respectively [19].

### Recruitment

A list of universities in Australia with accredited Bachelor of Nursing programs was identified from the Australian Health Practitioner Regulation Agency (APHRA) website. The first author sent an email invitation to the heads of nursing schools or the respective administration team of all identified universities who could facilitate permission to distribute the survey. After receiving approval from the universities interested in distributing the survey, the online survey link was sent through email. Each university nursing program then forwarded the invitation email, including the online survey link, to all final-year nursing students. The email also included a description of the study and information about confidentiality.

### Data analysis

#### Variables

The dependent variables in this study were the perception, knowledge, and attitude of final-year nursing students towards providing oral healthcare for older people. The independent variables included participants’ country of origin, geographic location, age, gender, previous experience with providing oral health, education about oral healthcare of older people and personal oral health behaviour.

#### Measurement of variables

First, the data were coded. The 5-point response categories on the perception, knowledge and attitude scales were scored as follows: 5 = strongly agree, 4 = agree, 3 = neither agree nor disagree, 2 = disagree, and 1 = strongly disagree for positive items. The negative items were reverse scored.

Descriptive data were analysed based on frequencies and percentages for categorical variables and means and standard deviations for continuous data. The categories *strongly agree* and *agree*, and *strongly disagree* and *disagree* on the Likert scale were combined for reporting the frequencies. Inferential statistics were employed to identify the factors associated with mean scores on the perception, knowledge, and attitude scales. With large sample sizes (> 30 or 40), the violation of the normality assumption should not pose significant concerns [29]; this means that parametric techniques can be used even when the data are not normally distributed [30, 31]. The association of various factors with students’ perception, knowledge, and attitude about providing oral healthcare was investigated with *t* tests (for two factors) and with ANOVA (for multiple factors). In the instance of ANOVA, if Levene’s test was significant, the Games-Howell post hoc test was performed, and if Levene’s test was not significant, the Turkey post hoc test was used to identify which samples were significantly different from one another. Spearman’s correlation coefficients tested the association between students’ perception of self-efficacy in providing oral healthcare and their knowledge of older people’s oral health. A *p-*value equal to or less than 0.05 was considered significant. SPSS software was used for the quantitative analysis of the study.

### Ethics approval

#### Ethics approval

was received from the Tasmanian Social Sciences Human Research Ethics Committee (ref no.H0020263). Participants were informed that participation was voluntary, and that data privacy and confidentiality would be maintained throughout the study. All participants understood that their consent was implied by their completion of the survey.

## Results

Of 36 universities delivering accredited Bachelor of Nursing programs, 16 universities consented to distribute the online survey link to their final-year students. Of 618 participants who assessed the survey and 560 participants who started the survey, a total of 416 participants completed the survey and their data are included in the analysis.

### Demographic characteristics

Of the 416 participants, most respondents (65%, n = 270) were born in Australia; most were female (92%, n = 381) and between the ages of 18 and 30 years (69%, n = 286). In terms of geographical location, 56% (n = 233) of participants lived in major cities, 32% (n = 134) of participants lived in a regional, rural, and remote area, and 10% (n = 40) had lived both in a major city and in a regional, rural, or remote area in the past ten years. In terms of geographical location of campuses, more than half of the students (51%, n = 212) were studying in a major city area, 38% (n = 156) in regional or rural or remote area, with the campus of the remainder either not specified or online. Table 1 summarises participants’ demographic characteristics.

**Table 1:** Background characteristics of participants (n = 416)

Variables	n	%
**Country of origin**		
Australia Other	270 146	64.9 35.1
**Geographical location of living in the past ten years**		
Most of time spent in a major city area Most of time spent in regional/rural/remote area Approximately equal time spent in a major city and a regional, rural, or remote area Not specified	233 134 40 9	56.0 32.2 09.6 02.2
**Geographical location of universities**		
Universities having campuses both in a major city and regional/rural/remote area	297	71.4
Universities having campuses only in a major city area	62	14.9
Universities having campuses only in regional/rural/remote area	57	13.7
**Geographical location of campuses**		
Major city area Regional or rural or remote area Online Not specified	212 156 31 17	51.0 37.5 07.5 04.1
**Age**		
18 years or less than 30 years 30 years or more than 30 years	286 130	68.8 31.3
**Gender**		
Female Male Others or prefer not to disclose	381 32 3	91.6 07.7 00.7

### Participants’ education and clinical preparation in providing oral healthcare

Table 2 presents data related to participants’ education, clinical preparation, and experience in providing oral healthcare to older people. Between a quarter and a third (28%, n = 116) of participants mentioned that they did receive oral health education and training in their previous education (e.g., certificate or diploma nursing course or during previous work experience). Most participants (80%, n = 332) had exposure to oral healthcare for older people during their practicum experience, mainly in hospitals (60%, n = 249) and residential age care (58%, n = 239).

**Table 2:** Participants’ education and clinical preparation in providing oral healthcare to older people (n = 416)

Variables	n	%
**Exposure to oral healthcare for older people during practicums**		
Yes	332	79.8
No	84	20.2
**Exposure sites during practicums**		
Hospital	249	59.9
Residential aged care	239	57.5
Independent living accommodation	20	04.8
A client’s home	33	07.9
**Exposure to oral healthcare activities during practicums**		
Oral health checks	99	23.8
Making dental referral	25	06.0
Oral healthcare planning	35	08.4
Oral hygiene tasks	317	76.2
Using an oral health product	232	55.8
Oral health care education	142	34.1
**Education or training in oral healthcare of older people in Bachelor of Nursing program**		
Yes	242	58.2
No	174	41.8
**Hours dedicated on the topic of oral healthcare for older people**		
< 1 h	146	35.1
1–2 h	80	19.2
> 3 h	16	03.8
**Type of oral health education received**		
Both practical and theoretical	169	40.6
Theoretical type	43	10.3
Practical only	30	07.2
**Education about oral health care products**		
Interdental cleaning aids	136	32.7
Mouthwashes	170	40.9
Saliva substitutes	67	16.1
Water hydration	142	34.1
Desensitising agents	44	10.6
Lip balms	155	37.2
Denture cleaning tablets and pastes	164	39.4
Denture adhesive pastes	87	20.9
Fluoride varnishes	40	9.61
Have not learned any of the above	24	5.8
**Clinical training in oral health checks**		
Presence and condition of natural teeth	125	30.0
Presence and condition of dentures	121	29.1
Condition of the lips	145	34.9
Condition of the gums and oral tissues	138	33.2
Condition of saliva	80	19.2
Condition of tongue	137	32.9
Oral pain	105	25.2
Oral cleanliness	161	38.7
Swallowing ability	172	41.3
Nutritional status/risk	159	38.2
Have not been trained in any of the above	22	5.2
**Oral healthcare education and training for older people before Bachelor of Nursing program**		
Yes	116	27.9
No	300	72.1
**Type of oral healthcare education and training received previously**		
Certificate or other diploma course	77	18.5
Work experience in older people’s care	58	13.9
Other	8	01.9
**Participants opinion on whether current nursing curriculum prepares nurses to provide effective oral health care to older people**		
Satisfied	183	44.0
Unsatisfied	233	56.0

As seen in Table 2, the experience of the 80% of participants (n = 332) who had exposure to oral healthcare activities focused mainly on oral hygiene tasks and the use of oral health products. Experience in making dental referrals and oral healthcare planning was limited. More than half of the participants (58%, n = 242) received education or training in oral healthcare for older people at university, but this education or training was frequently for less than one hour.

Only 41% (n = 169) of participants received both theoretical and practical oral health education. Of the participants (58%, n = 242) who received oral healthcare education and training, more than half reported that topics including saliva substitutes, desensitising agents, denture adhesive pastes, and fluoride varnishes were not covered. Detailed information can be found in Table 2. Similarly, participants often did not receive clinical training in crucial elements of oral health checks in their undergraduate nursing programs, including noting the condition of the lips and saliva, and the presence of oral pain (Table 2). More than half of the participants (56%, n = 233) believed that the current nursing curriculum did not prepare them to provide effective oral healthcare to older people (Table 2).

### Perception, knowledge, and attitude towards oral healthcare of older people

Table 3 presents the average mean scores of participants on the perception, knowledge, and attitude scales. The mean (± SD) scores on the 5-point perception and knowledge scales were 3.77 (± 0.75) and 3.77 (± 0.40), respectively. Participants’ mean score for attitude towards providing oral care was 4.47 (± 0.51). A mean score of four or above four (*agreed* or *strongly agreed* to positive statements) is considered to represent a perception of confidence, knowledge, and a favourable attitude towards providing oral healthcare to older people. A mean score below four is considered to represent a perception of lacking confidence, limited knowledge, and an unfavourable toward providing oral care. Mean scores showed that more than half of the participants felt they lacked confidence (55%, n = 229) and had limited knowledge about oral healthcare for older people (73%, n = 304); however, their attitude towards providing such care was favourable (89%, n = 369).

**Table 3:** Descriptive summary of average mean score of participants on perception, knowledge, and attitude scale

Variable	Mean scores	95% Confidence Interval for mean (Lower bound)	95% Confidence Interval for mean (Upper bound)	Standard deviation	N (%) of participants whose average mean score equal to or above four	N (%) of participants whose average mean scores below four
**Perception**	3.77	3.70	3.85	0.75	187 (45%)	229 (55%)
**Knowledge**	3.77	3.73	3.80	0.40	112 (26.9%)	304 (73.1%)
**Attitude**	4.47	4.42	4.50	0.51	369 (88.7%)	46 (11.3%)

*Negative items scored reversed

*Average mean score = Total sum of score acquired by participants in all perspective evaluating statements/ Number of Items in the perspective scale

#### Perception of self-efficacy in oral healthcare of older people

As presented in Table 4, approximately 80% of participants felt confident about their understanding of the factors affecting the oral health of older people and the relationship between oral and systematic health; 95% of participants felt that they were aware of effective daily oral healthcare activities. However, only 54% of participants were confident in supervising oral healthcare for older people and only 40% were confident in making timely and appropriate referrals for oral health assessment and care of older people.

**Table 4:** Frequency of response for each item evaluating participants’ perception of their self-efficacy to provide oral healthcare of older people

Statements	Strongly agree/agree N(%)	Neither agree or disagree N(%)	Disagree/ Strongly disagree N(%)
I understand the factors affecting the oral health of older people	337 (81%)	45 (10.8%)	34 (8.2%)
I am aware of effective daily oral health activities	396 (95.2%)	9 (2.2%)	11 (2.7%)
I am trained in making an appropriate, timely referral to a dentist for oral health assessment and care of older people when required	167 (40.1%)	95 (22.8%)	154 (37.1%)
I understand the relationship between oral and systemic health	321 (77.2%)	59 (14.2%)	36 (8.6%)
I am confident in training and supervising in providing oral healthcare to older people	224 (53.9%)	101 (24.3%)	91 (21.9%)

#### Knowledge of oral healthcare of older people

Table 5 presents the frequency of response for each item on the knowledge scale. Mixed results were found on this scale. Most participants were aware of factors affecting the oral health of older people, such as smoking (94%) and dry mouth problems (82%), whereas only 54% of participants knew that the presence of stringy saliva in the mouths of older people is not normal; 60% of participants were aware that fluoride toothpaste has greater benefits than non-fluoride toothpaste and 80% knew that a soft brush is better for cleaning teeth. However, only 31% were aware that brushing immediately after having carbonated drinks is not recommended. Around 20% of the participants were aware that using swabs containing lemon and glycerine to clean the mouths of older people is currently not recommended. Regarding denture knowledge, 75% of participants knew that dentures need to be brushed and 84% knew that dentures should be taken out of the mouth at night.

**Table 5:** Frequency of response for each item evaluating participants’ knowledge of oral healthcare of older people

Statements	Strongly agree/agree N(%)	Neither agree or disagree N(%)	Disagree/ strongly disagree N(%)
Smoking does not affect the oral health of older people with no natural teeth (F)	14 (3.3%)	11 (2.6%)	391 (94%)
Dry mouth problems are uncommon among older people (F)	48 (11.5%)	25 (6.0%)	343 (82.4%)
The presence of stringy saliva in the mouth of older people is normal (F)	67 (16.1%)	124 (29.8%)	225 (54.1%)
Fluoride toothpaste has no greater benefit than non-fluoride toothpaste for older people with natural teeth (F)	35 (8.4%)	131 (31.5%)	250 (60.1%)
A hard-bristled brush is better than a soft bristle brush for cleaning and removing plaque from older people’s teeth (F)	32 (7.7%)	85 (20.4%)	299 (70.9%)
Older people must brush their teeth immediately after having carbonated drinks (F)	103 (24.7%)	185 (44.5%)	128 ( 30.8)
Swabs containing lemon and glycerine should be used to clean the mouths of people who have no teeth (F)	108 (26%)	222 (53.4%)	86 (20.6%)
Denture cleaning solutions clean the dentures without you needing to brush the denture (F)	38 (9.1%)	66 (15.9%)	312 (75%)
Dentures should be taken out of the mouth at night (T)	348 (83.6%)	48 (11.5%)	20 (4.8%)
Cracked corners around the mouth can be treated with moisturiser (F)	198 (47.6%)	108 (26.0%)	110 (26.5%)
Bleeding gums while brushing do not require a dental referral (F)	22 (5.2%)	77 (18.5%)	317 (76.2%)
Older people without natural teeth only need a dental check-up when they have a problem (F)	27 (6.4%)	35 (8.4%)	354 (85.1%)
Cardiovascular problems can be associated with dental infections (T)	289 (69.5%)	112 (26.0%)	15 (3.6%)
People with diabetes have a higher risk of gum diseases and vice versa (T)	344 (82.7%)	60 (14.4%)	12 (2.9%)
Effective oral healthcare helps to prevent aspiration pneumonia (T)	281 (67.6%)	110 (26.4%)	25 (6%)

Items in the next section of the knowledge scale evaluated participants’ knowledge of making timely and appropriate referral. Around 76% of participants were aware that bleeding gums while brushing needed referral to a dentist, and 85% were aware that older people without natural teeth needed a routine dental check-up on a regular basis. However, only 27% were aware that cracked corners around the mouth needed more care than that provided by a moisturiser. The last section of the scale included items to check participants’ knowledge about the connection of oral health with general health. Most participants (70%) were aware that oral health is related to cardiovascular problems, 83% were aware that oral health is related to diabetes, and 68% were aware that oral health is linked to aspiration pneumonia.

#### Attitude towards providing oral healthcare to older people

Participants’ attitude towards providing oral healthcare to older people was favourable, as more than 90% of participants agreed or strongly agreed with most items on the attitude scale (Table 6). Nearly all (99%) believed that providing oral healthcare for older people is an important part of nursing care. Similarly, most participants (> 90%) showed interest in learning more about providing such oral healthcare and how nurses can work collaboratively with other health professionals in providing such care. In addition, almost 90% of participants were comfortable looking into older people’s mouths and assisting them with their daily oral hygiene.

**Table 6:** Frequency of response for each item evaluating participants’ attitude towards oral health care of older people

Statements	Strongly agree/agree N(%)		Neither agree or disagree N(%)	Disagree/ Strongly disagree N(%)
I believe the oral healthcare of older people is an important part of nursing care	412 (99%)	3 (0.7%)		1 (0.2%)
I am comfortable looking into the mouths of older people	361 (86.7%)	27 (6.5%)		28 (6.7%)
I am comfortable assisting older people with their daily oral hygiene	379 (91.1%)	23 (5.5%)		14 (3.4%)
I would like to learn more about the oral healthcare needs of older people	381 (91.6%)	30 (7.2%)		5 (1.2%)
I would like to learn more about how nurses can work with other health professionals in providing oral care to older people	396 (95.2%)	16 (3.8%)		4 (0.9%)

### Correlation of participants’ perception of self-efficacy in oral health care for older people and their knowledge of such care

A positive correlation was found between participants’ confidence in providing oral healthcare to older people and their perceived knowledge about such care (*r* = 0.13, *p* < 0.01).

### Factors associated with participants’ perception, knowledge, and attitude toward oral healthcare of older people

Results reported in Table 7 revealed statistically significant associations between participants’ mean perception scores of awareness of oral healthcare for older people and socio-demographic factors, including nationality [*t*= -3.41, *p* < 0.001], location in the past 10 years [*F* (3, 412) = 2.86, *p* < 0.05], experience in providing oral healthcare to older people [*t* = 4.52, *p* < 0.001], oral healthcare education in their current program [*t* = 3.32, *p* < 0.001], previous oral health education and training [*t* = 7.28, *p* < 0.001], and participants’ personal flossing habit [*F* (3, 412) = 5.67, *p* < 0.001]. Regarding participants’ knowledge of oral healthcare, factors such as age [*t* = -2.18, *p* < 0.05], gender [*t* = 2.28, *p* < 0.05], and experience in providing oral healthcare to older people [*t* = 2.87, *p* < 0.01] showed statistically significant associations. Participants’ attitudes towards providing oral healthcare were significantly associated with nationality [*t*= -3.54, *p* < 0.001], age [*t* = 2.10, *p* < 0.05], experience in providing oral healthcare [*t* = 2.65, *p* < 0.01], and previous oral health education and training [*t* = 3.59, *p* < 0.001].

**Table 7:** Factors associated with the participants’ perception, knowledge, and attitude towards oral healthcare of older people

Variable	N	Perception score		Knowledge score		Attitude score	
		**Mean (SD)**	**Significance test value**	**Mean (SD)**	**Significance test value**	**Mean (SD)**	**Significance** **test value**
**Nationality**							
1. Australia 2. Other	270 146	3.69 (0.75) 3.95(0.73)	t (414) = -3.41, p = < 0.001	3.80 (0.38) 3.72 (0.44)	t (414) = 1.73, p = 0.09	4.41 (0.50) 4.59 (0.49)	t (414) = -3.54, p = < 0.001
**Living location in the past ten years**							
1. Major city 2. Regional, rural, or remote area 3. Approximately equal time was spent in major and a regional, rural area 4. Not specified	233 134 40 9	3.72 (0.74) 3.79 (0.81) 4.03(0.63) 4.18 (0.42)	f (3,412) = 2.86, p = 0.04	3.77 (0.37) 3.80 (0.43) 3.67 (0.50) 3.65 (0.25)	f (3,412) = 1.48, p = 0.22	4.47 (0.50) 4.45 (0.55) 4.54 (0.44) 4.76 (0.32)	f (3,412) = 1.21, p = 0.31
		**Post hoc test result** 4 > 1*, 3 > 1*					
**University location**							
1. University having campuses both in major city and a regional or rural area 2. University having campuses only in major cities 3. University having campuses only in regional or rural area	297 62 57	3.74 (0.77) 3.79 (0.64) 3.95 (0.79)	f (2,413) = 1.93, p = 0.15	3.76 (0.40) 3.78 (0.42) 3.77 (0.41)	f (2,413) = 0.04, p = 0.96	4.49 (0.52) 4.52 (0.48) 4.34 (0.48)	f (2,413) = 2.15, p = 0.12
**Campus location**							
1. In a major city 2. In a regional or rural or remote area 3. Not specified	212 156 48	3.74 (0.65) 3.83 (0.81) 3.76 (0.96)	f (2,413) = 0.73, p = 0.48	3.76 (0.37) 3.76 (0.44) 3.85 (0.38)	f (2,413) = 1.12, p = 0.32	4.50 (0.49) 4.43 (0.51) 4.50 (0.59)	f (2,413) = 1.06, p = 0.34
**Age**							
1. 18 less than 30 years 2. 30 years or more	286 130	3.78 (0.72) 3.77 (0.81)	t (224.57) = 0.05, p = 0.96	3.74 (0.39) 3.83 (0.42)	t (414)= -2.18, p = 0.03	4.51 (0.46) 4.39 (0.59)	t (205.51) = 2.10, p = 0.04
**Gender**							
1. Female 2. Male	381 32	3.79 (0.73) 3.66 (0.99)	t (33.89) = 0.73, p = 0.47	3.79 (0.38) 3.55 (0.56)	t (33.43) = 2.28, p = 0.03	4.47 (0.50) 4.54 (0.60)	t (414)= -0.76, p = 0.44
**Provided oral healthcare to older people in your practicum experiences**							
1. Yes 2. No	332 84	3.86 (0.70) 3.45 (0.86)	t (414) = 4.52, p = < 0.001	3.80 (0.40) 3.66 (0.41)	t (414) = 2.87, p = 0.004	4.51 (0.48) 4.33 (0.59)	t (112.68) = 2.65, p = 0.009
**Received oral healthcare for older people education and training in Bachelor of Nursing program**							
1. Yes 2. No	242 174	3.88 (0.71) 3.63 (0.79)	t (414) = 3.32, p = < 0.001	3.76 (0.41) 3.78 (0.39)	t (414)= -0.41, p = 0.67	4.47 (0.50) 4.49 (0.53)	t (414)= -0.41, p = 0.68
**Received oral healthcare for older people education and training before current Bachelor of Nursing program**							
1. Yes 2. No	116 300	4.19 (0.62) 3.62 (0.74)	t (414) = 7.28, p = < 0.001	3.81 (0.43) 3.75 (0.39)	t (414) = 1.52, p = 0.12	4.60 (0.43) 4.42 (0.53)	t (253.19) = 3.59, p = < 0.001
**Oral healthcare behaviour** Brushing habit							
1. Twice daily 2. Once daily or less than daily 3. Thrice daily or more	330 60 26	3.80 (0.76) 3.62 (0.69) 3.83 (0.76)	t (2,413) = 1.50, p = 0.21	3.77 (0.41) 3.75 (0.37) 3.82 (0.46)	t (2,413) = 0.22, p = 0.80	4.48 (0.51) 4.39 (0.51) 4.57 (0.48)	t (2,413) = 1.30, p = 0.27
**Flossing habit**							
1. Less than daily 2. Once daily 3. Twice daily 4. Other	224 128 45 19	3.65 (0.73) 3.92 (0.75) 4.04 (0.81) 3.75 (0.69)	t (3,412) = 5.67, p = < 0.001	3.77 (0.37) 3.78 (0.41) 3.80 (0.54) 3.65 (0.36)	t (3,412) = 0.71, p = 0.54	4.44 (0.51) 4.53 (0.48) 4.55 (0.52) 4.32 (0.60)	t (3,412) = 1.74, p = 0.15
			**Post hoc test result** 2 > 1**, 3 > 1*				
**Usual reason of visiting a dentist**							
1. Regular check-up 2. Problem or other reason	239 177	3.80 (0.74) 3.75 (0.77)	t (414) = 0.60, p = 0.54	3.78 (0.41) 3.75 (0.40)	t (414) = 0.69, p = 0.49	4.49 (0.5) 4.46 (0.5)	t (414) = 0.56, p = 0.57
**Anxiety regarding visiting a dentist**							
1. No anxiety 2. Have anxiety	166 250	3.81 (0.77) 3.76 (0.74)	t (414) = 0.60, p = 0.55	3.79 (0.36) 3.76 (0.43)	t (414) = 0.82, p = 0.41	4.48 (0.51) 4.47 (0.51)	t (414) = 0.33, p = 0.73

*: p ≤ 0.05, **: p ≤ 0.01

## Discussion

The results from this study suggested that final-year nursing students studying at Australian universities, whether based in a major city or regional area, felt they lacked confidence and had limited knowledge of older people’s oral healthcare. Limited knowledge may have a negative impact on the quality of care these students will provide to older people once they graduate and enter the health workforce [32]. Previous research undertaken in several high-income nations has documented the poor understanding of nursing students about several crucial aspects of dental care [9, 13, 15, 33]. Similar results were found in the current study. However, in certain aspects of oral healthcare, participants in this study showed better knowledge in comparison to participants in other countries. In a New Zealand survey, only 38.7% of participants correctly answered denture care questions [9], whereas 70% of participants in the current study did so. A study done at Charles Sturt University in Australia reported that nursing students had a sound knowledge of periodontal problems but lacked the confidence to determine the causes of periodontal disease and distinguish between a healthy and diseased oral cavity [16]. Similarly, in the current study, only 54% of total participants were confident in supervising the provision of oral healthcare to older people and only 40% were confident about making timely appropriate referrals for oral health assessment and care of older people.

Another finding of the current study is that the nursing students’ attitude to providing oral healthcare to older people was positive. Compared to prior research performed in Japan and Turkey, Australian nursing students had a more positive attitude regarding providing oral care [14, 15]. In a survey done in Japan, just 43% of nursing students had a good opinion regarding dental health care practice, with only 40% of nursing students feeling the need to learn about denture care, and more than 50% of students thinking they did not need to know about general medicine or geriatrics in order to practise oral healthcare [15]. The majority of participants in the current study believed that oral healthcare was an important component of nursing care for older people and were eager to learn more about it, as well as how nurses can collaborate with other health professionals to provide effective oral healthcare to older people. These results were consistent with a study conducted in the US, which revealed that nursing students saw oral health care as a crucial component of effective nursing practice [13]. A previous study conducted in Australia showed similar results, where 91% of students agreed that oral health should be taught to nursing students [34]. A positive attitude regarding oral health will encourage nursing students to acquire oral health knowledge and bring improvement in oral health services delivery [35]. According to prior studies, nursing students who have a positive attitude towards oral care develop an interest in learning more about dental health and oral care [16, 33]. However, one study reported a lack of interest in learning about the oral healthcare of older people, which contrasts with the results of our study [15]. The authors of the prior study suggested interprofessional education and early clinical exposures in dental clinics and hospitals can develop interest among nursing students to provide oral healthcare to older people [15].

The results of this study revealed an underrepresentation of older people’s oral healthcare in nursing curricula which may be attributable to a low priority given to oral health care in nursing. This lack of oral health education in nursing curricula may explain why participants had limited knowledge in many crucial areas of oral healthcare for older people. Around 42% of participants in this study did not receive any educational component regarding oral healthcare for older people, and 35% reported that less than an hour was spent on this component. This is consistent with a survey of 112 schools of nursing in England that reported that 50% of the assessed schools had curricula which devoted less than an hour to older people’s oral health [36]. The current study reported that the curriculum often missed many important components of oral healthcare, including educating and encouraging older people to maintain their oral health, making appropriate and timely dental referrals, planning oral healthcare, and supervising caregivers to ensure evidence-based oral care is being provided to older people. These results aligned with studies exploring the views of nursing educators in Australia on the adequacy of learning and teaching oral health components in the nursing curriculum [18]. In a recent study, educators advocated including oral hygiene components of oral healthcare in the curriculum [37]. However, the results from the current study indicated that nursing students were lacking evidence-based knowledge to provide oral hygiene care to older people. Most participants were unaware that swabs containing lemon and glycerine should not be used to clean the mouths of people who have no teeth, as it can further exacerbate dry mouth conditions [38]. Therefore, it is vital to add more comprehensive information on oral healthcare to educate nursing students. There is a need for an express mandate from the Accreditation Council for Nursing Education to incorporate oral health education in nursing curricula as a prerequisite for accreditation, so that all universities may educate their students in oral health as integral to holistic care. It is important that components of oral healthcare that are crucial for nurses to understand are critically identified through interprofessional discussions. These identified components should be clearly stated in nursing education accreditation standards. This will ensure that the nursing curriculum is not overwhelmed with the education and training of nurses in the oral healthcare components which may be better handled by dental professionals. The concept of integrating oral health should not mean removing or replacing existing content, but rather expanding upon it [39]. Including oral anatomy and physiology in the anatomy and physiology module, or relating the relationship between oral and systemic health when instructing nursing students about diabetes, cardiovascular diseases, or pneumonia are some examples of how the integration of oral health can be done. Dental authorities should be invited to promote dental professionals’ visits in residential care and build collaboration with nurses and other allied health professionals to ensure oral healthcare delivery to older people. In this way nursing students can learn more about oral healthcare from dental and allied health professionals during their clinical placements. This can increase their interest and skills in this area.

This study identified several factors associated with the perception, knowledge, and attitude of nursing students towards oral healthcare of older people. A positive correlation was found between the perception of self-efficacy and knowledge in oral healthcare of older people which highlights the importance of knowledge on enhancing students’ confidence in carrying out oral healthcare activities. The perception scores of participants of Australian nationality were less than participants from other nations studying in Australian universities. In the research conducted in New Zealand, the nationality of nursing students was also associated with the knowledge of nursing students, but in this study, nationality was not found to be associated with knowledge though it was associated with attitude toward providing such care [9]. In relation to geographical location of living, it was found that the perception scores of participants who spent equal time in a regional or rural area and a major city area were higher than participants who spent most of their time in a major city only. Similarly, perception scores of participants who received education and training in oral healthcare for older people and had the experience of providing care to older people showed better confidence in their self-efficacy to provide oral healthcare to older people. One of the standout factors was experience in the oral healthcare of older people which was statistically significantly associated with all three dependent variables: perception, knowledge, and attitude. This is consistent with previous research [9]. Practicum experiences can be improved if registered nurses supervising these students have current, evidence-based knowledge and clinical skills in the oral healthcare of older people. There should be strong policies in the health and aged care facilities to ensure that oral hygiene products should be readily available to maintain regular oral healthcare. Role modelling of effective oral care at clinical placements will encourage nursing students to learn about the oral healthcare of older people and adopt these practices in their clinical practice. The other factor associated with both knowledge and attitude was age of participants, which has also been indicated in the literature [40]. In addition, knowledge scores were associated with gender, with female participants scoring more on the knowledge scale, and this has been noted in the previous study [41]. It is worth noting that the sample size in this study predominantly consists of females, which is consistent with the demographic composition of nursing students; perhaps it may have impacted the outcomes. Understanding the factors that influence knowledge and attitude can be valuable for educators as it enables them to design and incorporate oral health components into the nursing curriculum.

### Limitations

The main limitation of this study was the online self-report nature of information gathering. Response bias may limit the generalizability of findings, as nursing students with an interest in oral health and/or who are presently incorporating oral health into their practice may have been more likely to reply to the survey invitation. Furthermore, out of 36 universities, only 16 universities agreed to send the invitation to potential participants. This might lead to under/over-reporting of the level of knowledge and attitude of nursing students in Australia although almost half of accredited nursing programs participated. The internal validity of survey research can be hindered by the possibility of social desirability bias; however, the anonymity of the survey responses may have mitigated this limitation.

### Future research

Identification of oral healthcare knowledge gaps may facilitate the design of a curriculum related to the oral healthcare of older people in nursing. The current study findings can be used as a baseline to compare the knowledge and attitude of nursing students in Australia once oral healthcare for older people is effectively incorporated into the nursing curriculum. An observational study needs to be conducted to compare the self-reported highly favourable attitude of nursing students towards older people’s oral care and the attitude of nursing students in real-life practice.

## Conclusion

Nursing students from 16 universities, whether based in a major city or regional area, had a favourable attitude towards providing oral healthcare for older people, but their knowledge of such care was limited. The current study suggested that the Australian nursing curriculum is currently not preparing students for many essential oral healthcare topics, including conducting oral health checks, developing oral healthcare plans, and making dental referrals. A positive correlation was found between students’ confidence in delivering oral healthcare to older people and their perceived knowledge. Furthermore, the experience in providing oral healthcare to older people during practicum showed a statistically significant positive association with perception, knowledge, and attitude. It is strongly recommended that nursing programmes revise their curricula to promote oral healthcare, with more emphasis on oral health assessment as part of their regular general health assessment and improved curriculum content regarding the oral-systemic connection. Incorporating the oral healthcare component effectively in nursing curricula can be possible if the nursing accreditation authorities recognise oral healthcare of older people as an essential topic and promote interprofessional learning. Having evidence-based oral health knowledge and hands-on experience in providing oral healthcare to older people can improve the confidence of nursing students to deliver such care and the quality of care they provide to older people.

## Data Availability

The dataset(s) supporting the conclusions of this article is(are) included within the article.
